# Six Sigma revisited: We need evidence to include a 1.5 SD shift in the extraanalytical phase of the total testing process

**DOI:** 10.11613/BM.2020.010901

**Published:** 2020-02-15

**Authors:** Abdurrahman Coskun, Cristiano Ialongo

**Affiliations:** 1 Department of Medical Biochemistry, Acıbadem Mehmet Ali Aydınlar University, School of Medicine, Istanbul, Turkey; 2 Department of Physiology and Pharmacology, Sapienza University of Rome, Rome, Italy

**Keywords:** extraanalytical phase, shift, Sigma metric, Six Sigma, total testing process

## Abstract

The Six Sigma methodology has been widely implemented in industry, healthcare, and laboratory medicine since the mid-1980s. The performance of a process is evaluated by the sigma metric (SM), and 6 sigma represents world class performance, which implies that only 3.4 or less defects (or errors) *per* million opportunities (DPMO) are expected to occur. However, statistically, 6 sigma corresponds to 0.002 DPMO rather than 3.4 DPMO. The reason for this difference is the introduction of a 1.5 standard deviation (SD) shift to account for the random variation of the process around its target. In contrast, a 1.5 SD shift should be taken into account for normally distributed data, such as the analytical phase of the total testing process; in practice, this shift has been included in all type of calculations related to SM including non-normally distributed data. This causes great deviation of the SM from the actual level. To ensure that the SM value accurately reflects process performance, we concluded that a 1.5 SD shift should be used where it is necessary and formally appropriate. Additionally, 1.5 SD shift should not be considered as a constant parameter automatically included in all calculations related to SM.

## Introduction

The Six Sigma methodology represents an evolution in quality management that has been widely implemented in industry, healthcare, and laboratory medicine. Six Sigma is based on two important principles: 1) problem-solving approaches, such as define, measure, analyse, improve, and control (DMAIC) improvement cycle, and 2) quantitative statistical analysis. Through their combined use, the Six Sigma process aims to achieve very small output imprecision, such that 12 standard deviation (SD) units can be fit between the upper tolerance limit (UTL) and the lower tolerance limit (LTL) (*i.e.*, 6 SD can be fit between the target and the UTL/LTL). This is quantified through the sigma metric (SM), which can directly provide the number of defects *per* million opportunities (DPMO) (*1*).

**Figure 1:**
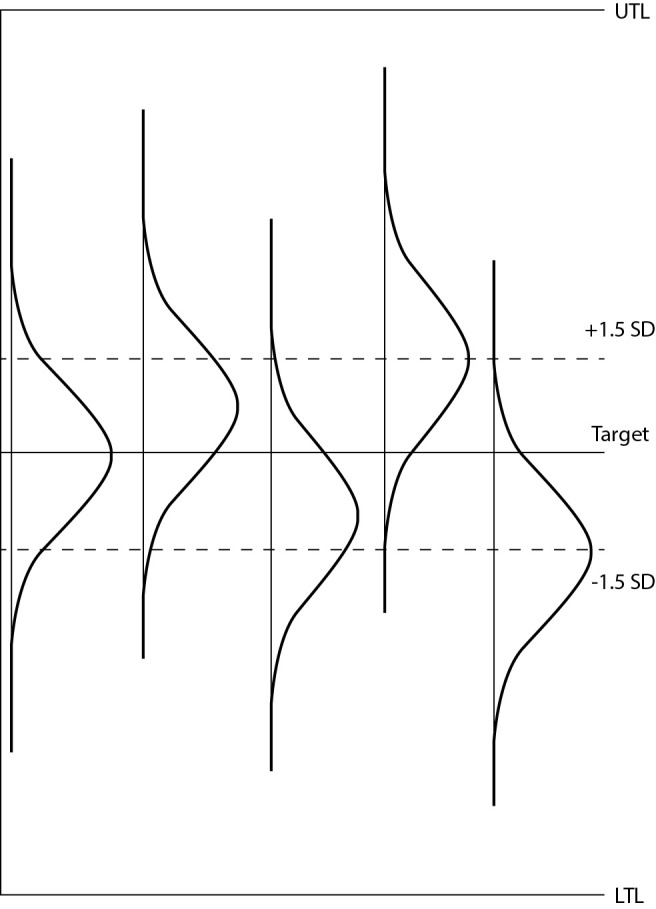
Shift of average of the process from its target. In routine practice, the process may deviate from its target. Therefore a 1.5 SD shift is included in the Sigma Metric calculation of the analytical phase of the total testing process. UTL - upper tolerance limit. LTL - lower tolerance limit. SD – standard deviation.

Quantitatively, the performance of ‘‘world-class’’ processes is 6 sigma, which implies that only 3.4 or less DPMO are expected to occur. Statistically however, 6 sigma corresponds to 0.002 DPMO rather than 3.4 DPMO. Since 0.002 parts-*per*-million corresponds to the area under the standard normal distribution curve that lays outside of the ± 6 SD distant from the mean. The reason for this difference is the introduction of a 1.5 SD shift to account for the random variation of the process around its target (Figure 1). In practice, the shift protects the process from underestimating the rate of non-compliances during the development stage. Consequently, a 6 sigma process is deemed to actually be 4.5 sigma in the routine phase (*2*). It should be noted that a 1.5 SD shift is part of the normal distribution graph and therefore should be applied to normally distributed data. If the distribution is not normal, then the SD cannot be used as measure of the shift of the average. However, in laboratory medicine it has been included in SM calculation for the extraanalytical phase. In this paper, we aim to 1) explain the reason for inclusion of a 1.5 SD shift in the analytical phase, and 2) show that inclusion of a 1.5 SD shift in the extraanalytical phase will cause great deviation in process performance from the actual level.

## Statistical basis of the 1.5 SD shift

The statistical basis of the 1.5 SD shift comes from the application of Shewhart control charts (Scc), like the X-bar and R chart, to monitor processes in real-time (*2*, *3*). In fact, the shift δ of a mean from its target that can be signalled by setting control limits to ± Z_α/2_ times the standard error is given by the equation below (*2*):
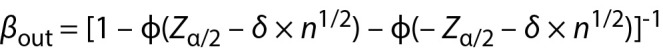
where β_out_ is the statistical power of the Scc used to detect the shift, Φ is the cumulative function of the standardized normal distribution, and n is the rational grouping size of observations taken to inspect the process.

It can be shown through a simple simulation that β_out_ is acceptable regardless of n (*i.e*. from 1 to 10) only when δ ≥ 1.5 SD for the process (*2*). From a standpoint of economic control, β_out_ is the average length of the production run (*i.e.* number of items) spent out of compliance before the shift δ is detected and the process is re-calibrated on its target. Therefore, there is a kind of “blind spot” in real-time process monitoring with Scc, for which 1.5 SD represents a correction factor (*2*).

Equation 1 suggests at least three reflections: 1) the application of the shift depends on the use of control charts to monitor the process, 2) the process must be stochastically normal (*i.e.* the output must be normally distributed over time) to be measured in terms of SD units, and 3) the actual size of the shift may be different (eventually smaller) than 1.5 SD. In the industrial field, where Six Sigma was originally developed, observation has indicated that the 1.5 SD shift is appropriate for manufacturing processes where long-term drift usually occurs (*3*). In clinical chemistry, where the Scc has been in use since the 1950s to control the analytical process (*i.e.* production of test results), the correspondence between the SM and the allowable Total Error (TEa) model has favoured the inheritance of the 1.5 SD shift (*4*). However, one may object that analytical processes are ideally less prone to long-time drift due to the higher frequency of their re-calibration and their ability to be externally controlled through participation in collaborative trials.

## Sigma metric in the extraanalytical phase

There is, however, another field in laboratory medicine – non-analytical processes – where the use of a 1.5 SD shift looks even more dubious. In 2000, Nevalainen *et al*. used the SM to rate the quality of processes of the extraanalytical phases (*i.e.* pre-analytical and post-analytical phases) of laboratories (*5*). In their work, the SM was obtained directly from the observed DPMO, using the “classical” industrial tables where the results of the conversion already included a 1.5 SD shift.

In the years that followed, the work of Nevalainen *et al.,* became the template for studies where a quality indicator (QI) is applied to estimate the performance of a process within the total testing process (TTP). Unfortunately, this seminal work disregarded that the calculation of SM as well as the application of the shift depends on the validity of Eq. 1. Thus, considering that neither of the extraanalytical process is likely to be normally distributed, nor are they monitored by Scc, there is concern regarding how the SM-to-DPMO conversion was accepted with no further investigation (*2*, *6*). The reason may lie in the application of a QI that produces a binomial measure (*i.e.* number of non-compliances), which corresponds to a probability distribution that becomes nearly normal if the number of the observations is adequately large (*i.e.* N > 20). Notably, this cannot be considered a sufficient condition, as the QI is a collated statistic of the output and not the output itself (*7*).

Remarkably, the error rate of the components within the TTP is heterogeneous, with the highest error rate seen in the pre-pre analytical phase and the lowest seen in the analytical phase. In the past decades, the analytical error rate has decreased significantly and consequently more than 90% of errors are now extraanalytic in nature (*8*). Therefore, a 1.5 SD shift may be reasonable only for the analytical phase, while including it as a dogma in other phases would unnecessarily inflate the error rate of the TTP. For example if the SM of post-analytical phase is 4, the DPMO is 32, not 6210. In extraanalytical phases we need evidence to include 1.5 SD as the shift of the process. From a pragmatic point of view, for example, it is not easy to find evidence to include 1.5 SD shift to pre-pre analytical phase. This should be of particular concern considering the heterogeneous nature of extraanalytical processes that may give rise to different long-term inflations of the error rate. For instance, in a totally automated laboratory the intra-laboratory turnaround time (TAT) depends on the operation of identical automated units that are subject to mechanical wear and maintenance, whereas the extra-laboratory TAT (*i.e.* patient-to-laboratory delivery time) mostly depends on the physical activity of human operators and their turnover (*8*). Thus, it is unwise to assume a priori that the error rate of different extraanalytical processes will inflate in exactly the same way over time.

## Conclusion

**Figure 2:**
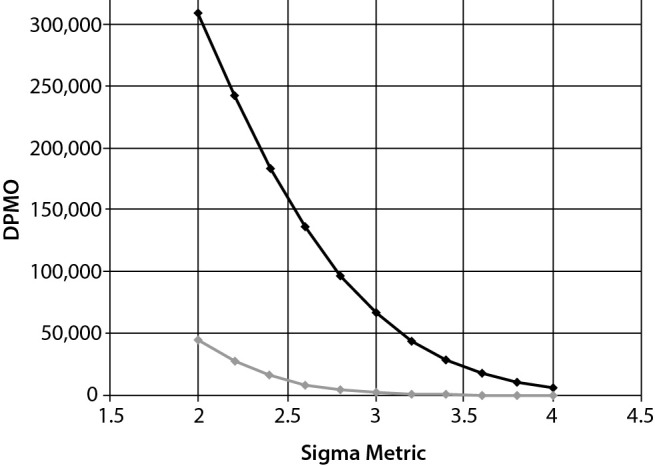
A nomogram to convert SM to DPMO and *vice versa* (gray curve). As shown in the Figure, a 1.5 SD shift creates a great deviation in DPMO (black curve). Inclusion of the 1.5 SD shift artificially increases the SM of the extraanalytical phase. DPMO - defects *per* million opportunities. SM – Sigma Metric. SD – standard deviation.

We have concluded that a 1.5 SD shift should no longer be considered a kind of universal constant to be applied to the SM across different fields regardless of their nature. In SM calculation, inclusion or exclusion of 1.5 SD shift should be evidence based, considering each analyte and process independently. To this end, a first step should be to discourage the use of tables where the “normal” SM with a 1.5 SD shift is provided. This is because the relationship between DPMO and SM is non-linear, so a small change in the SM can result in huge inflation of DPMO (Figure 2) (*9*). Furthermore, investigation of the dynamics of extraanalytical processes – especially if, and how much, they can drift – should be encouraged. Otherwise, the risk of continuing to use a constant 1.5 SD shift in laboratory medicine is that users will be misled, and the application of Six Sigma in this field will be blunted.

## References

[1] Coskun A Oosterhuis WP Serteser M Unsal I Sigma metric or defects per million opportunities (DPMO): the performance of clinical laboratories should be evaluated by the Sigma metrics at decimal level with DPMOs. Clin Chem Lab Med. 2016;54:e217–9. 10.1515/cclm-2015-1219 26760310

[2] Ialongo C Bernardini S Long story short: an introduction to the short-term and longterm Six Sigma quality and its importance in laboratory medicine for the management of extra-analytical processes. Clin Chem Lab Med. 2018;56:1838–45. 10.1515/cclm-2018-0310 29909405

[3] Harry MJ, Lawson JR. Six sigma producibility analysis and process characterization. Reading, MA: Addison-Wesley; 1992.

[4] Chesher D Burnett L Equivalence of critical error calculations and process capability index Cpk. Clin Chem. 1997;43:1100–1. 10.1093/clinchem/43.6.1100 9191580

[5] Nevalainen D Berte L Kraft C Leigh E Morgan T Evaluating laboratory performance on quality indicators with the six sigma scale. Arch Pathol Lab Med. 2000;124:516–9. 10747306 10.5858/2000-124-0516-ELPOQI

[6] Ialongo C Bernardini S Validation of the Six Sigma Z-score for the quality assessment of clinical laboratory timeliness. Clin Chem Lab Med. 2018;56:595–601. 10.1515/cclm-2017-0642 29040063

[7] Coskun A Six Sigma and laboratory consultation. Clin Chem Lab Med. 2007;45:121–3. 10.1515/CCLM.2007.023 17243930

[8] Plebani M Exploring the iceberg of errors in laboratory medicine. Clin Chim Acta. 2009;404:16–23. 10.1016/j.cca.2009.03.022 19302995

[9] Ialongo C Porzio O Giambini I Bernardini S Total Automation for the Core Laboratory: Improving the Turnaround Time Helps to Reduce the Volume of Ordered STAT Tests. J Lab Autom. 2016;21:451–8. 10.1177/2211068215581488 25882188

